# LABS score– a prognostic tool for FOLFOX4-treated advanced hepatocellular carcinoma and real-world efficacy: a single-center retrospective study

**DOI:** 10.1186/s12885-024-12040-z

**Published:** 2024-03-01

**Authors:** Jirapat Wonglhow, Patrapim Sunpaweravong, Chirawadee Sathitruangsak, Arunee Dechaphunkul

**Affiliations:** Division of Medical Oncology, Department of Internal Medicine, Faculty of Medicine, Prince of Songkla University, Songkhla, Thailand

**Keywords:** Hepatocellular carcinoma, Liver cancer, Chemotherapy, FOLFOX, Prognostic score

## Abstract

**Background:**

No widely used prognostic tool exists to demonstrate the benefit of oxaliplatin plus 5-fluorouracil/leucovorin (FOLFOX4) in patients with advanced hepatocellular carcinoma (HCC). We aimed to establish a prognostic score and demonstrate the real-world efficacy of FOLFOX4 chemotherapy in Thai patients.

**Methods:**

Between August 2017 and December 2021, we identified 58 FOLFOX4-treated patients with HCC. Overall survival (OS), progression-free survival (PFS), and objective response rate (ORR) were assessed. The prognostic score was constructed by stepwise Cox proportional hazards regression analysis to select variables for the best model with the lowest Akaike information criterion from all potential variables.

**Results:**

Forty-four patients (76%) received FOLFOX4 as first-line therapy. The ORR in the entire cohort was 8.6%, and the disease control rate was 29.3%. The PFS and OS were 3.7 and 4.8 months, respectively. Four clinically relevant variables were included in the new prognostic score to predict 6-month OS: L, the presence of lung metastasis; A, alcoholic cirrhosis; B, elevated total bilirubin level; and S, sorafenib-naïve status. Using the LABS score, patients were classified into low-, intermediate-, and high-risk groups, demonstrating OS values of 9.3, 4.2, and 2.1 months, respectively (*p* < 0.0001). The C-index and area under the receiver-operating characteristic curve of the score were 0.71 and 0.73, respectively.

**Conclusions:**

The proposed LABS score could discriminate patients who would derive benefit from FOLFOX4 chemotherapy. FOLFOX4 chemotherapy is an option for patients who cannot receive immunotherapy and targeted therapy, particularly those with a low-risk score. However, further validation of this model via larger cohorts is warranted.

**Supplementary Information:**

The online version contains supplementary material available at 10.1186/s12885-024-12040-z.

## Background

In 2020, hepatocellular carcinoma (HCC), the main type of primary liver cancer, was the sixth most frequently diagnosed cancer and the third leading cause of cancer-related death worldwide [1]. In South-Eastern Asian countries including Thailand, HCC is the major cause of cancer-related death among both men and women [1]. According to the estimation from the Thai National Cancer Register, the age-standardized incidence rates of liver and bile duct cancers in men and women are 33.9 and 12.9 per 100,000 per year, respectively [2]. The overall prognosis of HCC is considerably poor globally, and therefore incidence and mortality rates are roughly equivalent in different regions. In 2020, the mortality from and the estimated global incidence rate of liver cancer were 8.3 and 9.1 per 100,000 person-year, respectively [1].

The major causes of HCC, which are predominantly associated with cirrhosis, are hepatitis B virus (HBV) infection, hepatitis C virus (HCV) infection, alcohol use, and nonalcoholic fatty liver disease (NAFLD) [3]. HBV infection may correlate with the emergence of HCC in the absence of liver cirrhosis [3]. In Thailand, HBV infection is the leading risk factor for the development of HCC; however, HCV infection is more common in Western countries [4]. To date, as the population of patients with obesity is increasing, the risk of HCC in patients with NAFLD is rising worldwide, especially in some resource-rich regions [3, 4]. Despite the application of regular screening to detect early-stage HCC in high-risk populations, most patients with HCC especially in Asian countries, including Thailand, still frequently present with locally advanced or metastatic disease at the time of diagnosis [5, 6].

The mainstay of advanced HCC management is systemic treatment using molecularly targeted therapies with or without immune checkpoint inhibitors, which improves the overall survival (OS) of patients with advanced HCC; nevertheless, the survival benefit from chemotherapy remains uncertain [7–11]. According to the international guideline [7], the recommended first-line treatment of HCC comprises a combination of atezolizumab plus bevacizumab. Nevertheless, both immunotherapy and targeted therapy are limited used in Thailand as they are not readily affordable by patients. Hence, the best supportive care with or without palliative chemotherapy is a considerably important option for patients with good performance status in Thailand. FOLFOX4 chemotherapy is the most evidence-based regimen in advanced HCC. The EACH study, a phase III randomized control trial, directly compared the use of FOLFOX4 and doxorubicin as first-line treatments in patients with advanced HCC in Asian countries. There was a trend toward improvement in OS (mOS 6.4 months), progression-free survival (PFS), and response rate (RR) in FOLFOX4-treated patients than in doxorubicin-treated patients (mOS 4.97 months), although the survival benefits were not significantly different [12]. Nonetheless, a significant superior OS benefit of FOLFOX4 over doxorubicin was achieved in a subgroup of Chinese patients [13]. Additionally, FOLFOX4 chemotherapy was found to be more cost effective than sorafenib chemotherapy in China [14]. Therefore, the benefit of FOLFOX4 chemotherapy in Thai patients with advanced HCC should be explored.

Currently, there is no widely used prognostic tool to demonstrate the benefit of chemotherapy in patients with advanced HCC. Qin et al. proposed the only available prognostic nomogram using individual profiles of FOLFOX4-treated patients with advanced HCC [15]. Six variables were included in the prognostic models based on their clinical relevance: age, maximum tumor diameter, lymph node status, aspartate aminotransferase (AST) level, total bilirubin (TBIL) level, and alpha-fetoprotein (AFP) level. Although the prognostic nomogram was internally validated in Chinese patients, there was no evidence to support its generalizability. In addition, when applied to our patient cohorts, the nomogram could not clearly discriminate patients who would benefit from FOLFOX4 chemotherapy. (Wonglhow J, unpublished). Therefore, we aimed to establish a new prognostic score for FOLFOX4-treated patients with advanced HCC and to demonstrate the real-world efficacy of FOLFOX4 chemotherapy in Thai patients.

## Methods

### Study participants and procedure

This study was a retrospective, single-center study. Between August 2017 and December 2021, 58 patients received palliative chemotherapy with the FOLFOX4 regimen either in a first-line or later-line treatment setting at the Medical Oncology Service of Songklanagarind hospital. The data cutoff date was May 31, 2022. The inclusion criteria were as follows: (1) diagnosis of HCC with either typical imaging criteria (hypervascular pattern with arterial enhancement and rapid washout during the portal venous or delayed phase) or histological diagnosis; (2) advanced (failed/refractory to local treatment, metastatic, and/or recurrent) disease; and (3) exposure to at least one cycle of FOLFOX4 chemotherapy; and (4) age ≥ 18 years old.

We excluded patients without relevant information potentially required in the prognostic score, such as age, maximum tumor diameter, lymph node status, AST level, TBIL level, and AFP level.

Patient data were collected from the electronic medical record of the hospital information systems of Songklanagarind hospital. We collected baseline clinical (age at diagnosis, sex, body weight, body mass index [BMI], Eastern Cooperative Oncology Group [ECOG] performance status, presence of cirrhosis, causes of cirrhosis, Child-Pugh score, Barcelona clinical liver cancer [BCLC] stage, largest tumor diameter, number of liver tumors, and extrahepatic metastasis status) and laboratory parameters. HCC diagnosis and tumor burden, including tumor size and number, were determined by abdominal radiologists and recorded in the health information system. This study was reviewed and approved by the Ethics Committee of the Research Center of the Faculty of Medicine, Prince of Songkla University (approval number REC.66-246-14-1).

The FOLFOX4 chemotherapy regimen was administered as follows: a 2-h intravenous infusion of oxaliplatin (85 mg/m^2^) administered concurrently with leucovorin (200 mg/m^2^) on day 1, followed by an intravenous bolus of 5-fluorouracil (5-FU, 400 mg/m^2^); subsequently, 5-FU (600 mg/m^2^) was administered as a 22-h infusion immediately after the 5-FU bolus. Leucovorin and 5-FU administrations were repeated on day 2. The FOLFOX4 regimen was repeated at 2-week intervals until disease progression, death, an occurrence of unacceptable toxicity, or an indication of patient preference. In case of FOLFOX4 chemotherapy failure, other therapeutic regimens were considered depending on the patients’ performance status, preference, or affordability of the other regimens.

### Endpoints

The primary endpoint was to establish the prognostic score for FOLFOX4-treated patients with advanced HCC. The secondary endpoints were OS, PFS, objective RR (ORR), and disease control rate (DCR) of FOLFOX4 in patients with advanced HCC. OS was defined as the time from the first day of FOLFOX4 chemotherapy until death from any cause. PFS was defined as the time from the first day of FOLFOX4 chemotherapy until radiological tumor progression or death, whichever occurred first. The Response Evaluation Criteria for Solid Tumors were applied to define tumor progression and evaluate the RR. ORR was defined as the summation of the percentage of patients who achieved complete and partial response. DCR was defined as the summation of the percentage of patients who achieved complete response, partial response, and stable disease. Treatment response was assessed every 2–3 months using abdominopelvic and/or chest computed tomography. RRs were provided for all patients (intention-to-treat [ITT] analysis) and for assessable patients.

### Statistical analysis

For baseline characteristics, continuous variables were presented as median with interquartile range (IQR) or mean with standard deviation (SD), as appropriate, and categorical variables were presented as frequency with percentage. The prognostic score was constructed by stepwise Cox proportional hazards regression analysis to select variables for the best model with the lowest Akaike information criterion from all potential variables. Harrell’s concordance index (C-index) and the area under the receiver-operating characteristic curve (AUROC) were used to assess the score discrimination ability. Survival outcomes were estimated using Kaplan-Meier curves and compared via the log-rank test. Hosmer and Lemeshow goodness of fit test were used to demonstrate a good model calibration. All statistical analyses were performed with R software version 3.3.2 (R Foundation, Vienna, Austria). All *p*-values were two-sided, with *p* < 0.05 indicating statistical significance.

## Results

### Baseline characteristics

Forty-four (75.9%) and 14 (24.1%) patients received FOLFOX4 as first-line (first-line cohort) and second- to later-line (later-line cohort) treatments, respectively (Table 1). 82% of patients were men. The mean age at diagnosis was 54.5 years, and the median BMI was 22.2 kg/m^2^. Most patients had an ECOG score of 1 (82.8%). The majority of patients had cirrhosis (91.4%). 72% of patients had Child-Turcotte-Pugh (CTP) class A, and the rest has CTP class B. The most common cause of cirrhosis was HBV infection (72.4%). 31% of the patients had > 10 hepatic lesions, and 5.2% had infiltrative lesions. The mean diameter of the largest primary tumor was 11.1 cm. Approximately 88% of patients were diagnosed with BCLC stage C. Half of them had extra-hepatic metastasis with portal vein involvement. The most common sites of metastasis were the lungs (25.9%), lymph nodes (22.4%), and peritoneum (13.8%). Ten (17.2%) and 21 (36.2%) patients underwent tumor resection and transarterial chemoembolization, respectively. For patients who received prior systemic therapy (*n* = 14), sorafenib was the most common agent used (78.6%), followed by nivolumab (14.3%), doxorubicin (3.4%), regorafenib (3.4%), durvalumab plus tremelimumab per clinical trial (3.4%), and atezolizumab plus bevacizumab (1.7%). For baseline laboratory results, the median TBIL level, platelet count, international normalized ratio, and creatinine level were within normal limits. The median AST, ALT, and alkaline phosphatase levels were mildly elevated. The median AFP level was 6,056 ng/dL.

**Table 1:** Baseline characteristics

	Entire cohort (*n* = 58)	First-line cohort (*n* = 44)	Later-line cohort (*n* = 14)
**Setting**, n (%)			
First-line treatment Second-line treatment Third-line treatment Fourth-line treatment	44 (75.9) 10 (17.3) 2 (3.4) 2 (3.4)	44 (100) - - -	- 10 (71.4) 2 (14.3) 2 (14.3)
**Sex**, n (%)			
Female Male	10 (17.2) 48 (82.8)	7 (15.9) 37 (84.1)	3 (21.4) 11 (78.6)
**Age**, mean (SD), years	54.5 (9.1)	53.9 (9.1)	56.4 (9.3)
**BMI**, median (IQR), kg/m^2^	22.2 (19.8,24.7)	22.5 (20.1,24.7)	21.2 (19.9,24.2)
**Health care system**, n (%)			
CSMBS Social security Universal coverage Self-payment	7 (12.1) 7 (12.1) 40 (69.0) 4 (6.9)	4 (9.1) 7 (15.9) 32 (72.7) 1 (2.3)	3 (21.4) 0 (0) 8 (57.1) 3 (21.4)
**ECOG**, n (%)			
0 1 2	8 (13.8) 48 (82.8) 2 (3.4)	5 (11.4) 38 (86.4) 1 (2.3)	3 (21.4) 10 (71.4) 1 (7.1)
**Cirrhosis**, n (%)	53 (91.4)	40 (90.9)	13 (92.9)
**CTP score**, n (%)			
A B	42 (72.4) 16 (27.6)	31 (70.4) 13 (29.6)	11 (78.6) 3 (21.4)
**Etiology**, n (%) (* more than 1 answer)			
HBV HCV Alcohol NAFLD PSC	42 (72.4) 9 (15.5) 8 (13.8) 2 (3.4) 1 (1.7)	31 (70.5) 6 (13.6) 8 (18.2) 1 (2.3) 1 (2.3)	11 (78.6) 3 (21.4) 0 (0) 1 (7.1) 0 (0)
**Number of liver tumors**, n			
(%) 0 1–5 6–10 > 10 Infiltrative	7 (12.1) 27 (46.5) 3 (5.2) 18 (31.0) 3 (5.2)	4 (9.1) 21 (47.7) 1 (2.3) 15 (34.1) 3 (6.8)	3 (21.4) 6 (42.9) 2 (14.3) 3 (21.4) 0 (0)
**Maximum diameter of tumor**, mean (SD), cm	11.1 (6)	11.2 (5.7)	10.7 (7.1)
**Vascular involvement**, n (%)	33 (56.9)	27 (61.4)	6 (42.9)
**Ascites**, n (%)	7 (12.1)	5 (11.4)	2 (14.3)
**BCLC stage**, n (%)			
B C	7 (12.1) 51 (87.9)	5 (11.4) 39 (88.6)	2 (14.3) 12 (85.7)
**Extrahepatic metastasis**,			
n (%) 1 2 3 4	21 (36.2) 8 (13.8) 1 (1.7) 1 (1.7)	17 (38.6) 4 (9.1) 0 (0) 0 (0)	4 (28.6) 4 (28.6) 1 (7.1) 1 (7.1)
**Metastatic site**, n (%)			
Lymph node Lung Pleura Peritoneum Adrenal Bone Ovary Pancreas	13 (22.4) 15 (25.9) 2 (3.4) 8 (13.8) 2 (3.4) 2 (3.4) 1 (1.7) 1 (1.7)	7 (15.9) 8 (18.2) 0 (0) 6 (13.6) 1 (2.3) 1 (2.3) 1 (2.3) 1 (2.3)	6 (42.9) 7 (50.0) 2 (14.3) 2 (14.3) 1 (7.1) 1 (7.1) 0 (0) 0 (0)
**ALBI score**, n (%)			
1 2 3	12 (20.7) 39 (67.2) 7 (12.1)	8 (18.2) 30 (68.2) 6 (13.6)	4 (28.6) 9 (64.3) 1 (7.1)
**Lab**			
TBIL, median (IQR), mg/dL AST, median (IQR), U/L ALT, median (IQR), U/L ALP, median (IQR), U/L Albumin, mean (SD), g/dL Platelet count, median (IQR), /uL INR, median (IQR) Creatinine, median (IQR), mg/dL AFP, median (IQR), ng/dL	1.2 (0.7,1.7) 98 (57.8,230.5) 45 (30.8,77.0) 235(148.5,386.2) 3.5 (0.5) 209,000 (130750,260750) 1.2 (1.2,1.4) 0.8 (0.6,1) 6056(265,30103)	1.2 (0.7,1.7) 112 (61.5,280.5) 48 (29.8,76.0) 257.5(152.2,393.2) 3.5 (0.5) 211,000 (125000,269750) 1.3 (1.2,1.4) 0.8 (0.6,1) 5630.5(443,28734)	1.1 (0.6,1.9) 78.5 (49.5,109.5) 42.5 (33.0,71.0) 203 (77.5,256) 3.7 (0.5) 189,250 (139250,250000) 1.2 (1.1,1.2) 0.8(0.6,0.8) 24007.5(197,36309)
**Prior treatment**, n (%)			
Resection TACE RFA SBRT Doxorubicin Sorafenib Regorafenib Nivolumab Atezolizumab/Bevacizumab Durvalumab/Tremelimumab	10 (17.2) 21 (36.2) 5 (8.6) 2 (3.4) 2 (3.4) 11 (19.0) 2 (3.4) 2 (3.4) 1 (1.7) 2 (3.4)	4 (9.1) 14 (31.8) 5 (11.4) 2 (4.5) 0 (0) 0 (0) 0 (0) 0 (0) 0 (0) 0 (0)	6 (42.9) 7 (50) 0 (0) 0 (0) 2 (14.3) 11 (78.6) 2 (14.3) 2 (14.3) 1 (7.1) 2 (14.3)

SD, standard deviation; IQR, interquartile range; BMI, body mass index; CSMBS, civil servant medical benefit scheme; ECOG, Eastern Cooperative Oncology Group; CTP, Child-Turcotte-Pugh; HBV, hepatitis B virus; HCV, hepatitis C virus; NAFLD, nonalcoholic fatty liver disease; PSC, primary sclerosing cholangitis; BCLC, Barcelona clinic liver cancer; ALBI, albumin-bilirubin score; TBIL, total bilirubin; AST, aspartate aminotransferase; ALT, alanine aminotransferase; ALP, alkaline phosphatase; INR, international normalized ratio; AFP, alpha-fetoprotein; TACE, trans-arterial chemoembolization; RFA, radiofrequency ablation; SBRT, stereotactic body radiotherapy

### FOLFOX4 treatment, dose modification, and subsequent treatment

The treatment information is shown in S1 Table. The median numbers of FOLFOX4 cycles were 3, 2, and 5 in the entire, first-line, and later-line cohorts, respectively. Oxaliplatin dose reduction was started in 46.6% of the patients, whereas and 8.6% of the patients experienced 5-FU dose reduction initiation. Moreover, 75.9% and 15.5% had a dose reduction of oxaliplatin and 5-FU in the subsequent cycles, respectively. The major cause of treatment discontinuation was disease progression. Only 10.3% of FOLFOX4-treated patients with disease progression received later-line systemic therapy comprising doxorubicin (8.6%) and FOLFOX4 beyond progression (1.7%) (S2 Table).

### Prognostic factors

Initially, 18 clinically relevant potential variables were selected from the database considering both clinical and statistical aspects from the literature review: ECOG performance status, BMI, lymph node status, portal vein thrombosis, maximum tumor diameter, number of extrahepatic metastases, presence of lung metastasis, AST level, TBIL level, albumin level, platelet count, AFP level, HBV-related cirrhosis, HCV-related cirrhosis, alcoholic cirrhosis, prior surgical resection, line of FOLFOX4 treatment, and prior sorafenib treatment.

Univariate analysis showed that AST level, TBIL level, and alcoholic cirrhosis were significant baseline predictors of survival in patients with advanced HCC (S3 Table). The prognostic score was constructed by stepwise Cox proportional hazards regression analysis to select variables for the best model with the lowest Akaike information criterion from all potential variables. Four variables were subsequently entered into the model: L, the presence of lung metastasis; A, alcoholic cirrhosis; B, elevated TBIL level; and S, sorafenib-naïve status. Hazard ratios (HRs) and *p*-values of these variables are shown in Table 2.

**Table 2:** Cox proportional hazard model

Variables	Hazard ratio (95%CI)	*p*	Adjusted hazard ratio (95%CI)	*p*
Lung metastasis	1.37 (0.63,2.97)	0.430	2.44 (1.01,5.92)	0.048
Alcoholic cirrhosis	4.04 (1.71,9.57)	0.002	3.67 (1.44,9.35)	0.006
Elevated total bilirubin				
• Ref = < 2 mg/dL • 2–3 mg/dL • > 3 mg/dL	- 3.41 (1.14,10.2) 11.15 (4.08,30.43)	- 0.028 < 0.001	- 2.73 (0.85,8.78) 13.99 (4.83,40.54)	- 0.093 < 0.001
Sorafenib-naive	2 (0.7,5.73)	0.195	2.64 (0.8,8.69)	0.109

CI, confidence interval; Ref, reference

### Proposed prognostic score: LABS score

The LABS prognostic score (Table 3) was developed using the aforementioned four independent prognostic factors to predict the 6-month OS of FOLFOX4-treated patients with advanced HCC. A prognostic score was assigned to each predictor based on coefficient score from the calculation. The presence of lung metastasis, alcoholic cirrhosis, and sorafenib-naïve status were assigned 1 point each, whereas TBIL levels of 2–3 mg/dL and > 3 mg/dL were assigned 1 and 3 points, respectively. A total score was calculated by summing all the scores corresponding to each independent predictor; moreover, patients were classified into three groups based on the total score: low- (total score = 0), intermediate- (total score = 1–2), and high-risk groups (total score ≥ 3). The total score was used to estimate the 6-month OS probability in each risk group. A higher score implied a poorer survival outcome. The mOS in the low-, intermediate-, and high-risk groups were 9.3, 4.2, and 2.1 months, respectively. The 6-month OS probabilities were 61.3%, 40%, and 0% in the low-, intermediate-, and high-risk groups, respectively.

**Table 3:** LABS score

LABS Score	
Factors	Score
1. Presence of **L**ung metastasis	
No	0
Yes	1
2. Presence of **A**lcoholic cirrhosis	
No	0
Yes	1
3. Total **B**ilirubin level	
< 2 mg/dL	0
2–3 mg/dL	1
> 3 mg/dL	3
4. **S**orafenib-naïve status	
No	0
Yes	1
*Interpretation*: Low risk 0–1 Intermediate risk 2 High risk 3–6	*6-month Survival Probability* 61.3% 40.0% 0%

### Model performance

**Fig. 1:**
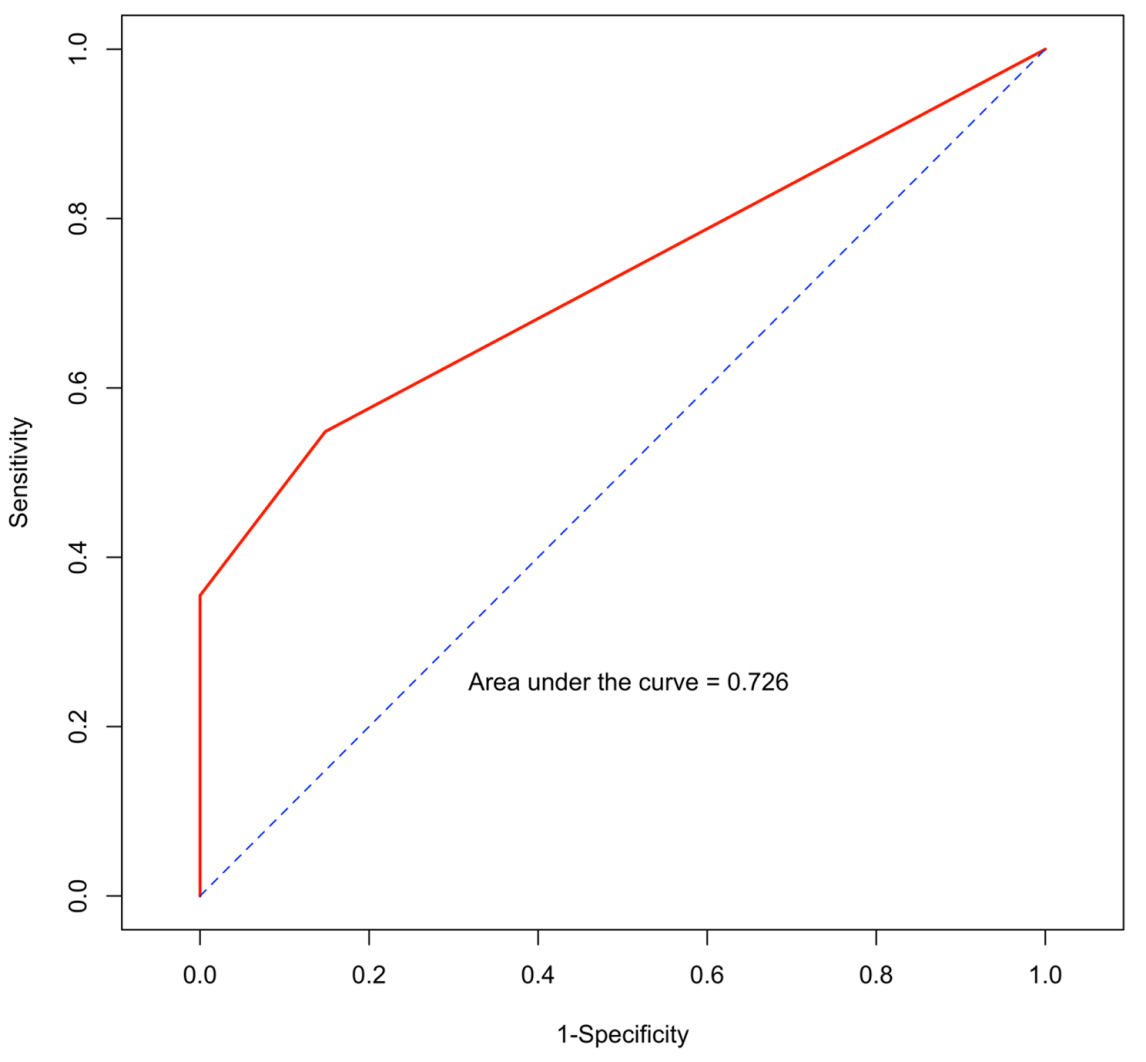
Area under receiver-operating characteristic curve of LABS score

The C-index of the model, which was internally validated using the bootstrap method with 1,000 iterations, for predicting the 6-month OS was 0.71. The AUROC of the LABS score was 0.73 (Fig. 1), which indicated a good performance for discrimination. The LABS prognostic score showed a non-significant outcome (*p* = 1) from the Hosmer and Lemeshow goodness of fit test, suggesting a good model calibration.

**Fig. 2:**
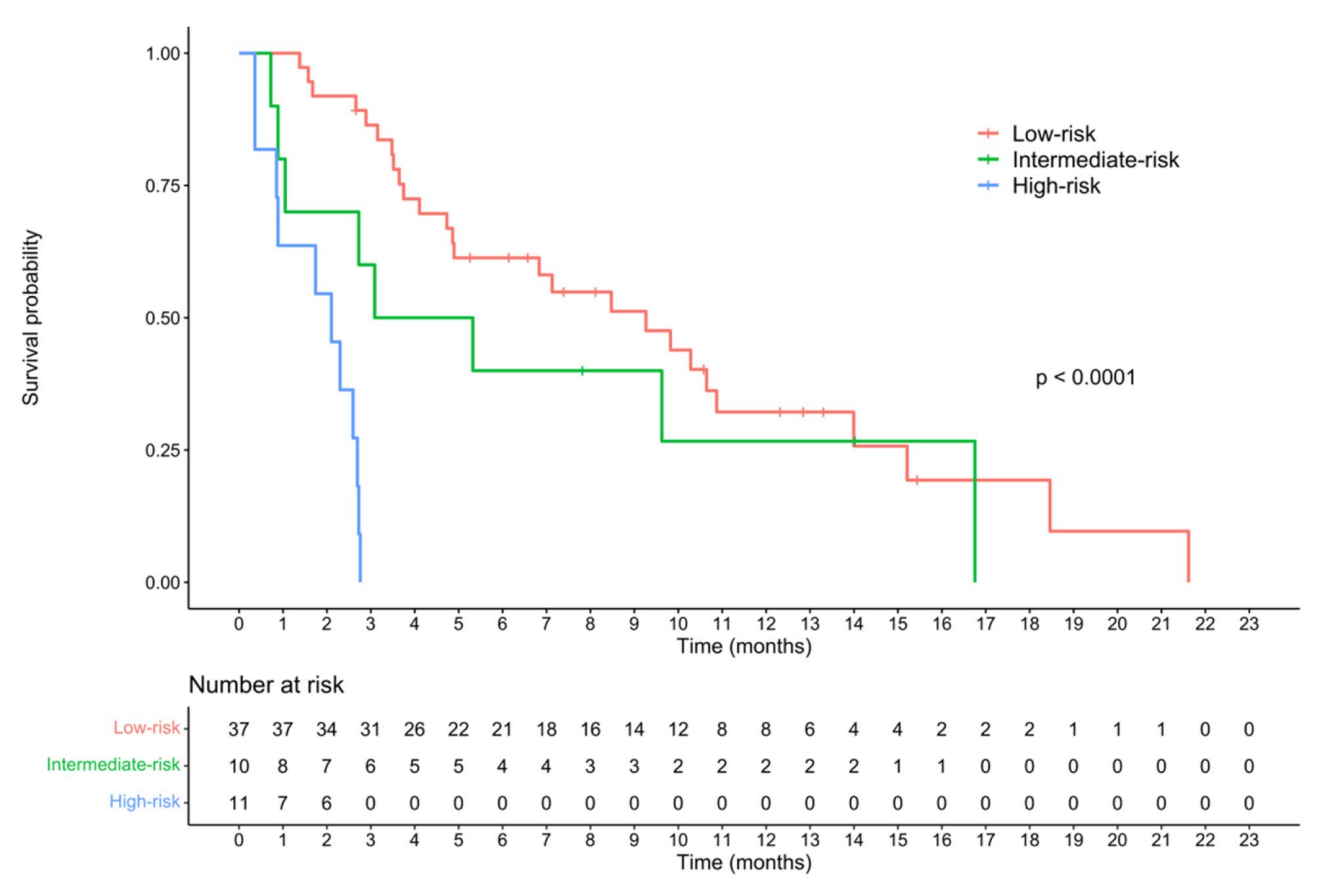
Kaplan-Meier curve of stratified survival based on the LABS score in FOLFOX4-treated patients with advanced hepatocellular carcinoma

The LABS prognostic score was used to divide patients into three groups, based on their 6-month survival probabilities predicted by the model. Furthermore, the Kaplan-Meier curve demonstrated a good discriminative ability of the score (*p* < 0.0001, Fig. 2).

### Real-world efficacies of FOLFOX4 chemotherapy

#### OS

**Fig. 3:**
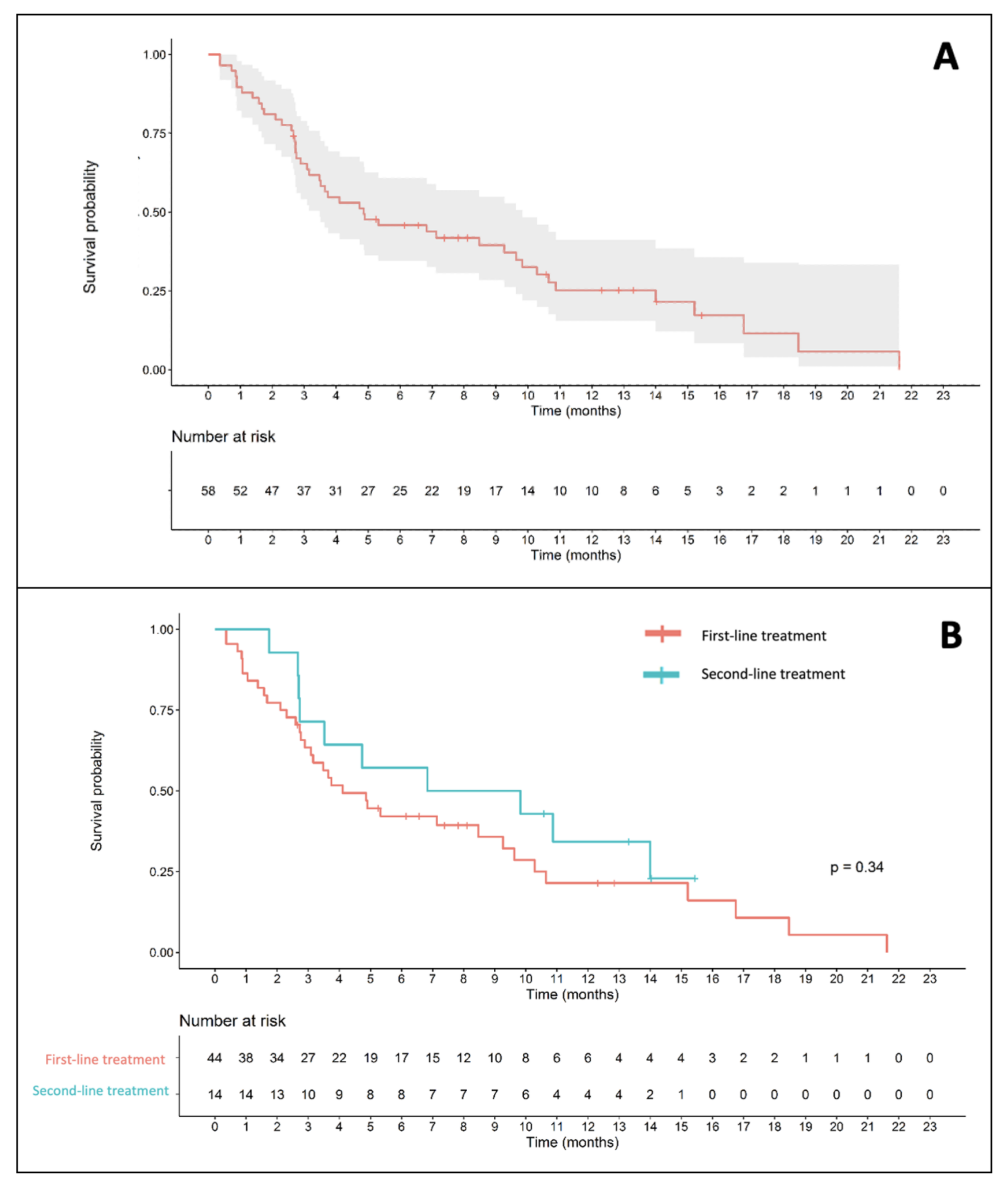
Overall survival (**A**) for entire cohort (**B**) by line of treatment

The median duration of follow-up was 4.8 months (IQR 2.66, 9.77). The mOS for the entire cohort was 4.9 months. When stratified by the line of treatment, the mOS for patients in the first- and later-line cohorts were 4.1 and 8.3 months, respectively (HR 0.71 (0.34, 1.44), *p* = 0.34) (Fig. 3).

### PFS

**Fig. 4:**
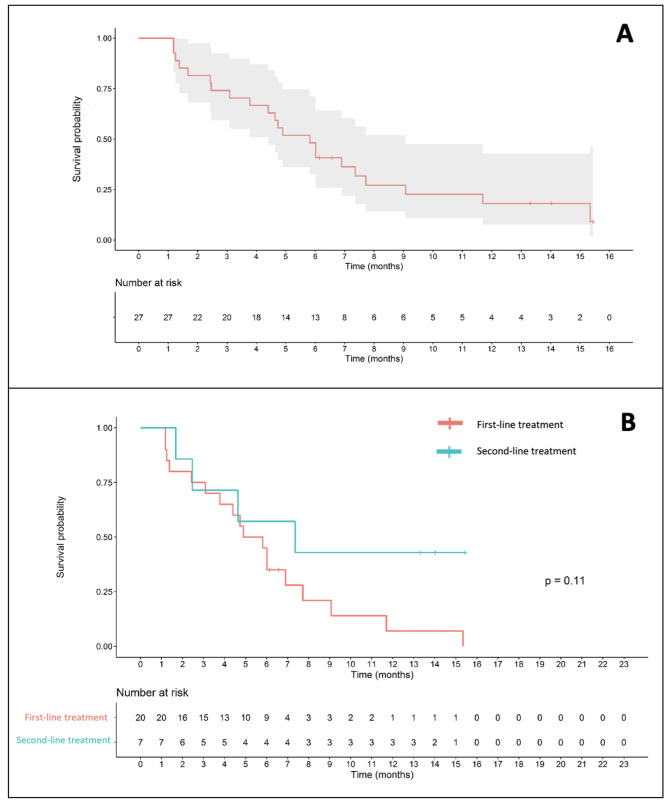
Progression-free survival (**A**) for entire cohort (**B**) by line of treatment

The median PFS for the entire cohort was 3.7 months. When stratified by the line of treatment, the median PFS of patients receiving FOLFOX4 as first-line treatment and second- to later-line treatment were 3.09 and 5.78 months, respectively (Fig. 4).

### RR

Radiological assessments were available for review in 50.7% of the 58 patients who received FOLFOX4 chemotherapy in the entire cohort. The ORRs for the entire cohort were 8.6% and 16.7% based on ITT and radiologically assessable analyses, respectively (Table 4). In the first-line cohort, 45.5% of patients had radiologically assessable data. The ORRs for the first-line cohort were 6.8% and 15.0% based on ITT and radiologically assessable analyses, respectively. The DCRs for the entire cohort were 29.3% and 56.7% based on ITT and radiologically assessable analyses, respectively.

**Table 4:** Treatment response

	Entire cohort (*n* = 58)	First-line cohort (*n* = 44)	Later-line cohort (*n* = 14)
Complete response, n (%)	0 (0)	0 (0)	0 (0)
Partial response, n (%)	5 (8.6)	3 (6.8)	2 (14.3)
Stable disease, n (%)	12 (20.7)	7 (15.9)	5 (35.7)
Progressive disease, n (%)	13 (22.4)	10 (22.7)	3 (21.4)
ORR per ITT, n (%)	5 (8.6)	3 (6.8)	2 (14.3)
ORR per assessable, n (%)	5 (16.7)	3 (15)	2 (20)
DCR per ITT, n (%)	17 (29.3)	10 (22.7)	7 (50)
DCR per assessable, n (%)	17 (56.7)	10 (50)	7 (70)

ORR, objective response rate; DCR, disease control rate

### Safety

Individual adverse events are detailed in Table 5. The most prevalent adverse events across the entire population were thrombocytopenia, AST elevation, and ALT elevation, all mostly categorized as grade 1–2. Notably, grade 3–4 adverse events exceeding 10% included neutropenia, AST elevation, and bilirubin elevation. Additionally, there was no statistically significant difference in adverse events between patients in the first-line cohort and those in the later-line cohort.

**Table 5:** Adverse events

AEs	Entire cohort (*n* = 58)		First-line cohort (*n* = 44)		Later-line cohort (*n* = 14)	
	All AEs n (%)	Grade 3-4 n (%)	All AEs n (%)	Grade 3-4 n (%)	All AEs n (%)	Grade 3-4 n (%)
Hematologic						
Neutropenia Leukopenia Thrombocytopenia Anemia	19 (32.8) 19 (32.8) 31 (53.4) 18 (31.0)	10 (17.2) 3 (5.2) 2 (3.4) 1 (1.7)	12 (27.3) 12 (27.3) 22 (50.0) 12 (27.3)	6 (13.6) 2 (4.5) 2 (4.5) 1 (2.3)	7 (50.0) 7 (50.0) 9 (64.3) 6 (42.9)	4 (28.6) 1 (7.1) 0 (0) 0 (0)
Non-hematologic						
Nausea Vomiting AST ALT TBIL Fatigue Diarrhea Sensory neuropathy	6 (10.3) 3 (5.2) 34 (58.6) 30 (51.7) 16 (27.6) 16 (27.6) 2 (3.4) 6 (10.3)	0 (0) 0 (0) 10 (17.2) 1 (1.7) 9 (15.5) 1 (1.7) 0 (0) 1 (1.7)	5 (11.4) 2 (4.5) 26 (59.1) 22 (50.0) 12 (27.3) 12 (27.3) 2 (4.5) 4 (9.1)	0 (0) 0 (0) 10 (22.7) 1 (2.3) 6 (13.6) 1 (2.3) 0 (0) 1 (2.3)	1 (7.1) 1 (7.1) 8 (57.1) 8 (57.1) 4 (28.6) 4 (28.6) 0 (0) 2 (14.3)	0 (0) 0 (0) 0 (0) 0 (0) 3 (21.4) 0 (0) 0 (0) 0 (0)

AEs, adverse events; AST, aspartate aminotransferase; ALT, alanine aminotransferase; TBIL, total bilirubin

## Discussion

This study establishes the newly proposed LABS score as a useful tool to predict the 6-month OS based on the following clinically relevant patient characteristics: L, the presence of lung metastasis; A, alcoholic cirrhosis; B, elevated TBIL level; and S, sorafenib-naïve status. Patients in the low-risk group had the best prognosis, followed by those in the intermediate- and high-risk groups. Furthermore, the new prognostic LABS score clearly discriminated patients who would benefit from FOLFOX4 treatment.

In Thailand, immunotherapy and targeted therapy are limited used as these are not readily affordable by patients. In addition, only sorafenib can be provided as first-line treatment in patients under the Civil Servant Medical Benefit Scheme. Therefore, the best supportive care with or without palliative chemotherapy is the only available treatment option for the rest of the patients with good performance statuses. Although there is no substantial evidence to support a survival benefit from chemotherapy [7–11], FOLFOX4 is the most evidence-based regimen in patients with HCC [12, 13]. Thus, it is crucial to establish a tool based on clinical characteristics to guide oncologists in defining which group of patients would benefit from FOLFOX4 chemotherapy.

The variables identified in our model included the presence of lung metastasis, alcoholic cirrhosis, elevated TBIL level, and sorafenib-naïve status. The prognostic score was developed using these variables to predict survival outcomes in each group of patients receiving FOLFOX4. Patients in the low-risk group achieved the highest benefit from FOLFOX4 chemotherapy (6-month survival probability, 61.3%), followed by those in the intermediate-risk group, who achieved a moderate benefit (6-month survival probability, 40%). In contrast, patients in the high-risk group had the worst prognosis (6-month survival probability, 0%), indicating that FOLFOX4 chemotherapy may not provide any additional OS benefit compared with the best supportive care alone. Hence, our findings supported the predictive benefit of the LABS score in selecting suitable patients for FOLFOX4 chemotherapy.

Pulmonary metastasis is the most common extrahepatic metastasis in patients with HCC. In a previous study, approximately 11.2–25.5% of patients with HCC had lung metastasis at the time of initial diagnosis, and it was a major cause of mortality among these patients [16]. According to a large retrospective cohort study, the 1- and 3-year survivals after diagnosis for HCC patients with pulmonary metastasis were 10.8% and 2.3%, respectively. Furthermore, patients treated with chemotherapy had a median survival of 4.0 months compared with 1.0 months for those who did not receive chemotherapy [16]. Hence, our result suggested that pulmonary metastasis should be considered an important prognostic factor for HCC. Moreover, the incidence of pulmonary metastasis (25.9%) in our study was comparable with that of the abovementioned study, indicating that it was a substantial prognostic factor for HCC, with an adjusted HR of 3.69 for OS.

Cirrhosis is a crucial factor affecting the survival of patients with HCC [17–19]. Decompensated cirrhosis worsens patients outcome [20] due to cirrhosis itself and the reduced tolerability to chemotherapy among chemotherapy-treated patients [21]. Furthermore, the etiology of cirrhosis might affect survival outcomes among patients with HCC. Notably, a large database study reported that patients with HBV-associated HCC had better survival outcomes than those with other HCC etiologies [22]. Patients with alcoholic cirrhosis had a higher risk of mortality and decompensated cirrhosis than those with chronic HCV infection or NAFLD-related cirrhosis; moreover, patients with alcoholic cirrhosis had higher rates of acute-on-chronic liver failure and hepatic encephalopathy than those with HBV-related cirrhosis [22, 23]. Of the eligible patients in our study, 49% and 13.8% had HBV infection and alcoholic cirrhosis, respectively. The adjusted HR of OS for patients with alcoholic cirrhosis was 9.37.

HCC patients with elevated TBIL levels had a worse prognosis than those with normal levels. The incidences of portal vein thrombosis, tumor multifocality, and high AFP levels were also increased in patients with elevated bilirubin levels, regardless of the primary tumor size. A previous study reported an association between bilirubin levels and indices of HCC aggressiveness [24]. Our study showed that elevated TBIL level was a potent factor for OS with an adjusted HR of 20.22.

Generally, HCC is resistant to chemotherapy, probably due to microenvironmental properties, including tissue stiffness and oxygen concentration [25]. The tumor microenvironment can potentially affect drug metabolism and subsequent treatment response to common therapeutic modalities, such as chemotherapy, targeted therapy, and immunotherapy [25–28]. An in-vitro study found a correlation between differential therapeutic response and cytochrome p450-3A4 (CYP3A4) enzyme expression level regulation under the influence of tissue stiffness and oxygen concentration variation. HCC cells with higher baseline CYP3A4 enzyme expression levels exhibited a cirrhosis-dependent increase in doxorubicin chemoresistance. In contrast, HCC cells with lower CYP3A4 enzyme expression levels showed a decrease in doxorubicin chemoresistance in response to increased microenvironmental stiffness. Furthermore, the addition of sorafenib lowered the dose of doxorubicin required to induce significant levels of cell death [25]. These previous study findings might potentially explain our study findings, wherein patients who received sorafenib before chemotherapy had better survival outcomes than sorafenib-naïve patients.

To the best of our knowledge, only one study has developed a nomogram to indicate the survival outcomes of FOLFOX4-treated patients with advanced HCC [15]. However, no external validation has been hitherto performed to support the use of the nomogram. The information used in the nomogram was extracted from the database of the EACH study, which is a phase III randomized control trial [12, 13]. The nomogram included six variables: age, maximum tumor diameter, lymph node status, AST level, TBIL level, and AFP level. TBIL level was the only variable present in both our nomogram and in that of the EACH study. Furthermore, our patients had more high-risk baseline characteristics (S4 Table) than patients in the EACH trial. Specifically, more patients in our study had liver cirrhosis, CTP class B, alcoholic liver disease, BCLC stage C, ascites, larger maximum tumor diameter, and higher AFP levels. Thus, these findings may highlight the differences between the clinical trial and real-world situations where data cannot be entirely applied. Our study established a new, easy-to-use prognostic tool, with a good model performance of discrimination. Our prognostic tool was acceptable and demonstrated superiority with C-index of 0.71 compared to the previous staging systems: BCLC (0.67) and American Joint Commission on Cancer 7th edition (0.63) [19, 29, 30]. Additionally, it was comparable to the C-index of the prognostic nomogram developed by Qin et al. (0.75) [15].

The PFS in our study was comparable with that of a previous study; however, the OS and ORR were lower in our study than in the previous study [11] (S5 Table). This finding might be explained by a few factors. First, the lower survival in our cohort might have resulted from the different baseline characteristics in relation to a real-world setting; the patients had more heterogeneous and high-risk features. In our study, cirrhosis was the most important factor related to the treatment efficacy, as patients with cirrhosis had worse prognoses than those without cirrhosis; specifically, one-quarter of our study patients had CTP class B. Second, it well-established that the chemotherapy dose intensity affects treatment response and survival outcomes in patients with cancer. In our study, 50% of patients started with a dose reduction of oxaliplatin, and 75% had a dose reduction of oxaliplatin in subsequent cycles, as the cirrhotic liver might not tolerate a standard chemotherapy dose intensity. Lastly, although the subsequent lines of treatment constituted a considerable factor leading to survival prolongation, no patient in our cohort received any approved standard treatment at the time of disease progression (Table 3). The ORR based on ITT analysis in our study was 8.6%, which was comparable to that of the EACH study (8.15%) [12] but lower than that of other previous studies (15–20%) [11]; moreover, the disease control rate in our study was 26%, which was lower than those of all other studies (40–60%) [11]. However, the ORR and disease control rate based on radiologically assessable analysis in our study were 16.7% and 56.7%, respectively. Therefore, this finding might have been affected by an unexpectedly low level of radiological assessment in our study, wherein only half of the patients underwent computed tomography for response evaluation.

In terms of safety, our study revealed no new safety concerns associated with FOLFOX treatment in HCC. When compared to the safety data from the EACH trial [12], our study indicated a lower incidence of hematologic toxicity, encompassing neutropenia, thrombocytopenia, and anemia. This discrepancy in toxicity rates could potentially be attributed to the administration of a lower chemotherapy dose in our study. Specifically, approximately 43% of patients initiated the first cycle of FOLFOX with a dose reduction of oxaliplatin, and throughout the course of treatment, 75% of the patients experienced a dose reduction of oxaliplatin. This observed pattern of dose reductions may contribute to the reduced incidence of hematologic toxicity, highlighting the potential impact of dose management strategies on treatment tolerability.

A key strength of our study was developing the novel prognostic tool, which demonstrated a good model performance for discrimination. This can aid clinicians in using real-world clinical data to select patients who might benefit from FOLFOX4 chemotherapy. Furthermore, our study showed the efficacy of FOLFOX4 chemotherapy in patients with advanced HCC having heterogeneous and real-world characteristics. Therefore, our prognostic tool is expected to be more applicable and generalizable in real-world settings.

Our study had some limitations. First, it was a single-center study with a small sample size. Second, missing data were unavoidable due to the retrospective nature of the study. Lastly, the radiological assessment level was unexpectedly low, which could affect the actual ORR and PFS outcomes.

## Conclusions

The proposed LABS score can clearly discriminate patients with advanced HCC who would benefit from FOLFOX4 chemotherapy. It is a potentially useful and feasible tool to guide oncologists in treatment decision making. FOLFOX4 chemotherapy should be considered an option for patients who cannot undergo immunotherapy and targeted therapy, especially those in a low-risk group based on LABS score assessment. However, large-scale studies are warranted to validate this model.

### Electronic supplementary material

Below is the link to the electronic supplementary material.

Supplementary Material 1

## Data Availability

The datasets used and/or analysed during the current study are available from the corresponding author on reasonable request.

## Abbreviations

HCC: Hepatocellular carcinoma
HBV: Hepatitis B virus
HCV: hepatitis C virus
NAFLD: Nonalcoholic fatty liver disease
OS: Overall survival
mOS: Median overall survival
PFS: Progression-free survival
RR: Response rate
AST: Aspartate aminotransferase
TBIL: Total bilirubin
AFP: Alpha-fetoprotein
BMI: Body mass index
ECOG: Eastern Cooperative Oncology Group
BCLC: Barcelona clinical liver cancer
ORR: Objective response rate
DCR: Disease control rate
ITT: Intention-to-treat
IQR: Interquartile range
SD: Standard deviation
C-index: Concordance index
AUROC: Area under the receiver-operating characteristic curve
CTP: Child-Turcotte-Pugh
HR: Hazard ratio
CYP3A4: Cytochrome p450-3A4

## References

[1] Sung H, Ferlay J, Siegel RL, Laversanne M, Soerjomataram I, Jemal A (2021). Global cancer statistics 2020: GLOBOCAN estimates of incidence and mortality worldwide for 36 cancers in 185 countries. CA Cancer J Clin.

[2] Imsamran W, Pattatang A, Supaattagorn P, Chiawiriyabunya I, Namthaisong K, Suwanrungruang K (2018). Cancer in Thailand: vol. IX, 2013–2015.

[3] Marrero JA, Kulik LM, Sirlin CB, Zhu AX, Finn RS, Abecassis MM (2018). Diagnosis, staging, and management of hepatocellular carcinoma: 2018 practice guidance by the American Association for the study of Liver diseases: Marrero et al. Hepatology.

[4] Chitapanarux T (2015). Risk factors for the development of hepatocellular carcinoma in Thailand. J Clin Transpl Hepatol.

[5] Chonprasertsuk S, Vilaichone RK (2017). Epidemiology and treatment of hepatocellular carcinoma in Thailand. Jpn J Clin Oncol.

[6] Sithinamsuwan P (2000). Review of 336 patients with hepatocellular carcinoma at Songklanagarind Hospital. World J Gastroenterol.

[7] National Comprehensive Cancer Network. Hepatobiliary cancers (Version 4.2021)[Internet].[cited 2021 Aug 28]. Available from:https://www.nccn.org/professionals/physician_gls/pdf/hepatobiliary.pdf.

[8] Thai Association for the. Study of the Liver.Thailand guideline for management of hepatocellular carcinoma 2021.2021.

[9] Finn RS, Qin S, Ikeda M, Galle PR, Ducreux M, Kim TY (2020). Atezolizumab plus Bevacizumab in unresectable hepatocellular carcinoma. N Engl J Med.

[10] Llovet JM, Hilgard P, de Oliveira AC, Forner A, Zeuzem S, Galle PR (2008). Sorafenib in advanced hepatocellular carcinoma. N Engl J Med.

[11] Qin S, Gong X (2016). Progression of systemic chemotherapy with oxaliplatin-containing regimens for advanced hepatocellular carcinoma in China. Hepat Oncol.

[12] Qin S, Bai Y, Lim HY, Thongprasert S, Chao Y, Fan J (2013). Randomized, multicenter, open-label study of oxaliplatin plus fluorouracil/leucovorin versus doxorubicin as palliative chemotherapy in patients with advanced hepatocellular carcinoma from Asia. J Clin Oncol.

[13] Qin S, Cheng Y, Liang J, Shen L, Bai Y, Li J (2014). Efficacy and safety of the FOLFOX4 regimen versus doxorubicin in Chinese patients with advanced hepatocellular carcinoma: a subgroup analysis of the EACH study. Oncologist.

[14] Zhang P, Wen F, Li Q (2016). FOLFOX4 or sorafenib as the first-line treatments for advanced hepatocellular carcinoma: a cost-effectiveness analysis. Dig Liver Dis.

[15] Qin S, Zhang X, Guo W, Feng J, Zhang T, Men L (2017). Prognostic nomogram for advanced hepatocellular carcinoma treated with FOLFOX 4. Asian Pac J Cancer Prev.

[16] Feng J, He Y, Wan J, Chen Z (2020). Pulmonary metastases in newly diagnosed hepatocellular carcinoma: a population-based retrospective study. HPB.

[17] Tham J, Goh TL, Barclay S, Priest M, Forrest E, Fraser A (2021). P221 non-cirrhotic vs cirrhotic HCC: comparison between patient characteristics, aetiology and outcomes. Gut.

[18] Yen YH, Cheng YF, Wang JH, Lin CC, Wang CC (2021). Characteristics and etiologies of hepatocellular carcinoma in patients without cirrhosis: when East meets West. PLoS ONE.

[19] Tandon P, Garcia-Tsao G (2009). Prognostic indicators in hepatocellular carcinoma: a systematic review of 72 studies: prognostic indicators in hepatocellular carcinoma. Liver Int.

[20] Zipprich A, Garcia-Tsao G, Rogowski S, Fleig WE, Seufferlein T, Dollinger MM (2012). Prognostic indicators of survival in patients with compensated and decompensated cirrhosis. Liver Int.

[21] Edeline J, Raoul JL, Vauleon E, Guillygomac’h A, Boudjema K, Boucher E (2009). Systemic chemotherapy for hepatocellular carcinoma in non-cirrhotic liver: a retrospective study. World J Gastroenterol.

[22] Brar G, Greten TF, Graubard BI, McNeel TS, Petrick JL, McGlynn KA (2020). Hepatocellular carcinoma survival by etiology: a SEER-Medicare database analysis. Hepatol Commun.

[23] Marot A, Henrion J, Knebel JF, Moreno C, Deltenre P (2017). Alcoholic liver disease confers a worse prognosis than HCV infection and non-alcoholic fatty liver disease among patients with cirrhosis: an observational study. PLoS ONE.

[24] Carr BI, Guerra V, Giannini EG, Farinati F, Ciccarese F, Rapaccini GL (2014). Association of abnormal plasma bilirubin with aggressive hepatocellular carcinoma phenotype. Semin Oncol.

[25] Özkan A, Stolley DL, Cressman ENK, McMillin M, DeMorrow S, Yankeelov TE (2021). Tumor microenvironment alters chemoresistance of hepatocellular carcinoma through CYP3A4 metabolic activity. Front Oncol.

[26] Sas Z, Cendrowicz E, Weinhäuser I, Rygiel TP (2022). Tumor microenvironment of hepatocellular carcinoma: challenges and opportunities for new treatment options. Int J Mol Sci.

[27] Lohitesh K, Chowdhury R, Mukherjee S (2018). Resistance a major hindrance to chemotherapy in hepatocellular carcinoma: an insight. Cancer Cell Int.

[28] Tian X, Yan T, Liu F, Liu Q, Zhao J, Xiong H (2022). Link of sorafenib resistance with the tumor microenvironment in hepatocellular carcinoma: mechanistic insights. Front Pharmacol.

[29] Llovet JM, Bru C, Bruix J. Prognosis of hepatocellular carcinoma: the BCLC staging classification. Semin Liver Dis.1999;329– 38. 10.1055/s-2007-1007122 10518312

[30] Vauthey JN, Lauwers GY, Esnaola NF, Belghiti J, Mirza N, Curley SA et al. Simplified staging for hepatocellular carcinoma. J Clin Oncol.2002;1527–36. 10.1200/JCO.2002.20.6.1527 11896101

