# Supplementary material for: LABS score– a prognostic tool for FOLFOX4-treated advanced hepatocellular carcinoma and real-world efficacy: a single-center retrospective study
Source: BMC Cancer. 2024 Mar 1;24:281. doi: 10.1186/s12885-024-12040-z (PMC10908120; doi:10.1186/s12885-024-12040-z)
Supplement: Supplementary file 1 — Supplementary Material 1 [file 12885_2024_12040_MOESM1_ESM.docx]

**Supplementary Information**

**S1 Table** Treatment information

|  | **Entire cohort**  **(n=58)** | **First-line cohort**  **(n=44)** | **Later-line cohort**  **(n=14)** |
| --- | --- | --- | --- |
| **Number of cycles**, median (IQR) | 3 (1.2,6.8) | 2 (1,6) | 5 (2.2,11.8) |
| **Oxaliplatin**  Started dose, n (%)   - Full dose - First-level reduction - Second-level reduction   Dose reduction, n (%) | 31 (53.4)  25 (43.1)  2 (3.4)  44 (75.9) | 24 (54.5)  18 (40.9)  2 (4.5)  31 (70.5) | 7 (50)  7 (50)  0 (0)  13 (92.9) |
| **5-FU**  Started dose, n (%)   - Full dose - First-level reduction - Second-level reduction   Dose reduction, n (%) | 53 (91.4)  4 (6.9)  1 (1.7)  9 (15.5) | 41 (93.2)  2 (4.5)  1 (2.3)  5 (11.4) | 12 (85.7)  2 (14.3)  0 (0)  4 (28.6) |
| **Discontinuation**, n (%)  Complete course  Death  Liver decompensation  Patient preference  Progressive disease  Poor performance status (declined)  Referred to nearby hospital  Toxicity | 2 (3.4)  9 (15.5)  10 (17.2)  2 (3.4)  22 (37.9)  7 (12.1)  1 (1.7)  1 (1.7) | 1 (2.3)  8 (18.2)  7 (15.9)  2 (4.5)  18 (40.9)  6 (13.6)  1 (2.3)  0 (0) | 1 (7.1)  1 (7.1)  3 (21.4)  0 (0)  4 (28.6)  1 (7.1)  0 (0)  1 (7.1) |

IQR, interquartile range.

**S2 Table** Subsequent treatment

|  | **Entire cohort**  **(n=58)** | **First-line cohort**  **(n=44)** | **Later-line cohort**  **(n=14)** |
| --- | --- | --- | --- |
| Subsequent therapy, n (%) | 7 (12.1) | 7 (15.9) | 0 (0) |
| Systemic therapy, n (%) | 6 (10.3) | 6 (13.6) | 0 (0) |
| Chemotherapy, n (%)   - Doxorubicin - FOLFOX4 (beyond progression) | 5 (8.6)  1 (1.7) | 5 (11.4)  1 (2.3) | 0 (0)  0 (0) |
| Palliative radiotherapy, n (%) | 3 (5.2) | 1 (2.3) | 2 (14.2) |

FOLFOX, folinic acid (leucovorin), 5-fluorouracil, and oxaliplatin.

**S3 Table** Univariate and multivariate analysis of potential variables

| **Variables** | **Univariate** | | **Multivariate** | |
| --- | --- | --- | --- | --- |
|  | **HR (95%CI)** | ***p*** | **HR (95%CI)** | ***p*** |
| Maximum tumor diameter | 1.05 (0.52,2.12) | 0.892 | - | - |
| Vascular involvement | 1.49 (0.71,3.11) | 0.289 | - | - |
| LN involvement | 1.55 (0.69,3.48) | 0.287 | - | - |
| Number of extrahepatic metastases | 1.52 (0.58,4) | 0.397 | - | - |
| Lung metastasis | 1.37 (0.63,2.97) | 0.43 | 2.44 (1.01,5.92) | 0.048 |
| HCV-related cirrhosis | 1.84 (0.79,4.29) | 0.156 | - | - |
| HBV-related cirrhosis | 0.47 (0.23,0.97) | 0.041 | - | - |
| Alcoholic cirrhosis | 4.04 (1.71,9.57) | 0.002 | 3.67 (1.44,9.35) | 0.006 |
| AST level | 2.18 (0.97,4.93) | 0.061 | - | - |
| AFP level | 1.56 (0.67,3.64) | 0.298 | - | - |
| TBIL level | 11.15 (4.08,30.43) | < 0.001 | 13.99 (4.83,40.54) | < 0.001 |
| Albumin level | 0.55 (0.27,1.15) | 0.112 | - | - |
| Platelet count | 1.08 (0.51,2.29) | 0.847 | - | - |
| BMI | 0.83 (0.3,2.31) | 0.72 | - | - |
| ECOG PS | 3.39 (0.63,18.13) | 0.153 | - | - |
| Line of FOLFOX4 treatment | 0.63 (0.26,1.54) | 0.312 | - | - |
| Previous sorafenib  (No vs. Yes) | 2 (0.7,5.73) | 0.195 | 2.64 (0.8,8.69) | 0.109 |
| Previous resection | 0.53 (0.19,1.53) | 0.243 | - | - |

CI, confidence interval; HR, hazard ratio; BMI, body mass index; ECOG PS, Eastern Cooperative Oncology Group performance status; LN, lymph node; HBV, hepatitis B virus; HCV, hepatitis C virus; TBIL, total bilirubin; AST, aspartate aminotransferase; AFP, alpha-fetoprotein.

**S4 Table** Differences in baseline characteristics between our cohorts and the EACH study

|  | **Entire cohort**  **(n=58)** | **First-line cohort**  **(n=44)** | **EACH study**  **(n=187)** |
| --- | --- | --- | --- |
| Age (mean), years | 54.5 | 53.9 | 50 |
| Male, % | 82.8 | 84.1 | 90.2 |
| Cirrhosis, % | 91.4 | 90.9 | 55.4 |
| Child-Turcotte-Pugh score, %  A  B | 72.4  27.6 | 70.4  29.6 | 88.6  11.4 |
| Etiology, %  HBV  HCV  Alcohol | 72.4  15.5  13.8 | 70.5  13.6  18.2 | 92.9  4.97  - |
| Number of liver tumors, %  0  1 – 5  6 – 10  >10  Infiltrative type | 12.1  46.5  5.2  31.0  5.2 | 9.1  47.7  2.3  34.1  6.8 | 3 (1-11)  Median (IQR) |
| Maximum tumor size, cm (SD) | 11.1 (6) | 11.2 (5.7) | 7.85 (4.75-11.7)  Median (IQR) |
| AFP, ng/dL | 6056 | 5630 | 1312 |
| BCLC, %  B  C | 12.1  87.9 | 11.4  88.6 | 21.2  78.8 |
| Extrahepatic metastasis, % | 53.4 | 47.7 | 56.5 |
| Portal vein thrombosis, % | 56.9 | 61.4 | 60.9 |
| Ascites, % | 12.1 | 11.4 | 3.3 |
| TBIL, mg/dL | 1.2 | 1.2 | 0.91 |
| AST, U/L | 98 | 112 | 38 |

HBV, hepatitis B virus; HCV, hepatitis C virus; SD, standard deviation; IQR, interquartile range; AFP, alpha-fetoprotein; BCLC, Barcelona clinic liver cancer; ALBI score, albumin-bilirubin score; TBIL, total bilirubin; AST, aspartate aminotransferase.

**S5 Table** Differences in efficacy of chemotherapy with FOLFOX or XELOX in clinical trials enrolling patients with advanced hepatocellular carcinoma

| **Study** | **Design** | **No of patients** | **Treatment** | **DCR (%)** | **ORR (%)** | **TTP/ PFS** | **Median OS** | **Cirrhosis (%)** | **CTP A/B (%)** | **2L setting (%)** |
| --- | --- | --- | --- | --- | --- | --- | --- | --- | --- | --- |
| **Qin S et al.** | Open-label, randomized, Phase III | 184/187 | FOLFOX4/ doxorubicin | 52.2 | 8.15 | 2.9 months | 6.4 months | 55.7 | 88.6/11.4 | 20.6 |
| **Qin S et al.** | Open-label, randomized, Phase III | 140/139 | FOLFOX4/ doxorubicin | 47.1 | 8.6 | 2.4 months | 5.7 months | 55.4 | 90/10 | 20.7 |
| **Yang L et al.** | Single-arm | 77 | FOLFOX4 | 55.6 | 4.2 | 2.7 months | 6.1 months | - | 77.9/22.1 | - |
| **Zhou et al.** | Single-arm | 20 | FOLFOX | 60.0 | 20.0 | 2.2 months | 5.0 months | - | 68.8/31.2 | - |
| **Yang L et al.** | Clinical observation | 31 | CAPOX | 42.9 | 7.1 | 2.9 months | N/A | 54.8 | 80.6/19.4 | - |
| **Yin Z et al.** | Clinical observation | 20/20 | CAPOX/ FOLFOX6 | 55.0 | 5.0 | 2.1 months | 9 months | - | - | - |
| **He SL et al.** | Single-arm | 32 | CAPOX | 62.5 | 21.9 | 4.2 months | 9.2 months | - | - | - |
| **Wang F et al.** | Clinical observation | 13 | FOLFOX4/  CAPOX | 61.5 | 15.4 | 3.9 months | 8.0 months | - | - | 100.0  post sorafe-nib |
| **Our study** | Retrospec-tive | 58 | FOLFOX4 | 29.3 (ITT)  56.7 (assess-able) | 8.6 (ITT)  16.7 (assess-able) | 3.7 months | 4.86 months | 91.4 | 72.4/27.6 | 24.0 |

FOLFOX, folinic acid (leucovorin), 5-Fluorouracil, and oxaliplatin; CAPOX, capecitabine and

oxaliplatin; DCR, disease control rate; ORR, objective response rate; TTP, time to progression; PFS, progression-free survival; OS, overall survival; CTP, Child-Turcotte-Pugh; ITT, intention-to-treat.
